# Coping better with health problems after a visit to the family physician: associations with patients and physicians characteristics

**DOI:** 10.1186/s12875-018-0712-y

**Published:** 2018-02-07

**Authors:** Christine Cohidon, Pascal Wild, Nicolas Senn

**Affiliations:** 1 Institute of Family Medicine, Department of Ambulatory Care and Community Medicine, University of Lausanne, Rue du Bugnon 44, CH-1011 Lausanne, Switzerland; 2 Institute for Work and Health, Lausanne University and Geneva University, Lausanne, Switzerland; 3 INRS - National Research and Safety Institute, Vandoeuvre les Nancy, France

**Keywords:** Coping better, Family medicine, Patients’ characteristics, Physicians’ characteristics - practices’ characteristics–multilevel analysis

## Abstract

**Background:**

Good patient experience is recognized as an important component of a strong primary care system. Among the dimensions related to experience in family medicine, the ability to cope better with health problems is considered to be a measure of the quality of a consultation with a family physician (FP). The objective is to identify factors related to patients, physicians and practice, associated with patients’ ability to cope better with their health problems after a family medicine consultation.

**Methods:**

The data stemmed from the Swiss part of the Quality and Costs of Primary Care (QUALICOPC) study, an international cross sectional survey aiming to compare quality, cost and equity in primary care. In Switzerland, a random sample of 199 FPs and 1791 patients participated. The negative answer to the question: “After this visit, I feel I can cope better with my health problems” was modeled using multilevel logistic regressions.

**Results:**

Difficulty to cope better with health problems was positively associated with the following: younger age (OR: 1.58, 95% CI [1.03–2.41]), cultural aspects related to the Swiss area of language (French speaking people declared higher inability than German and Italian ones), presence of chronic disease (OR: 1.54 95% CI [1.00–2.39]). Conversely an intermediate number (1–4) of visits during the last 6 months (OR: 0.37 95% CI [0.23–0.62]) and the satisfaction with the physician (OR: 0.18 95% CI [0.08–0.44]) are negative predictors of the patient inability to cope better with his health problems. A self-reported effort-reward imbalance at work (OR: 0.64 95% CI [0.41–1.00]) was the only predictive FP characteristic (negatively associated).

**Conclusions:**

Although the design of the study does not allow causal inference, this study showed that the predictors of patient difficulties to cope better with health problem are mainly centered on the patients’ characteristics. The patient-physician relationship both in terms of quality and frequency of visits is probably also important. Organizational practice characteristics do not seem to play a major role but stress at work among physicians should be further investigated.

## Background

Besides clinical indicators, patient experience of care is nowadays considered as one of the key components in the quality measurement of a primary care system [1, 2]. Patient feeling of confidence and ability to cope with his health problems after a medical visit (through the global concept of enablement) is, among many dimensions used to assess patient experience, considered as an interesting patient-reported outcome measure (PROM) [3–6]. In a recent review of literature, Frost defines the concept of enablement as “an intervention by which the health care provider recognizes, promotes and enhances a patient’s ability to manage their own health” [3]. The studies about predictive factors of enablement are scarce and sometimes contradictory [7]. However, male gender, older people, absence of longstanding illness and belonging to ethnic minority groups were found to be associated with a better patient enablement in several studies [7–9]. With the exception of longer consultations [7, 9, 10], few characteristics related to the physician or the organization of the consultation have been found to potentially play a role; and most of them concerned the patient-physician relationship [3, 7, 11–13]. Frost et al. underlined that further research is needed both to consolidate the results about patients’ predictors and to explore further physicians’ factors [3]. Moreover, we hypothesize that other characteristics related to practice organization, such as group practice, working with paramedical disciplines, and weekly workload, could also play a role [14]. Lastly, according to some studies, physician exposure to stress at work could impact the quality of delivered care and patient experience [15, 16]. However to our knowledge, the relationship between family physician job stress factors and patient coping with health trouble has never been investigated. One study has explored potential links between patient enablement and FP burnout and reported negative results [17].

The objective of the present study is to describe the relationship between patient feeling of coping better with his health problem after a family medicine consultation and both patient personal characteristics (sociodemographic and health features) and his family physician features (sociodemographic, personal and organizational features).

## Methods

### Study population

Data stemmed from the Swiss participation in the QUALICOPC study, a cross-sectional European survey coordinated by the Nivel Institute from Netherlands. This survey conducted in 2012 aimed to analyze and compare how primary health care systems in different countries perform in terms of quality, costs, and equity [18]. Details about the QUALICOPC study have been published elsewhere [18, 19]. Surveys were held among family physicians (FPs) in 31 European countries. In each country, a random nationally representative sample of 200 GPs was drawn. Only one physician per practice or health centre was eligible to participate. In Switzerland, the participating physicians (199) stemmed from a random sample of FPs stratified by canton. The representativeness in terms of gender, age, and rural/urban distribution was cross-checked against national statistics and considered satisfactory [20]. In each practice, nine patients (randomly drawn) filled in a Patient Experience questionnaire about the consultation which had just occurred. The resulting sample of patients consisted of 1791 persons.

The Swiss data collection took place between January and June 2012 and was conducted by the Department of ambulatory care and community medicine of the University of Lausanne.

### Data

The patient questionnaire was self-administrated at the practice. Patient enablement was explored through the following question: “after this visit, I feel I cope better with my health problems” (YES /NO/DO NOT KNOW), which is one of the 6 items of the Patient Enablement Instrument (PEI) questionnaire [9, 21]. The QUALICOPC study was developed using a multidimensional approach; in that context, using entire questionnaires to assess each different concept was unrealistic. The questionnaires were developed by experts consensus rounds basing upon a bibliometric search [9, 19]. Sociodemographic characteristics of the patients were collected: sex, age, place of birth, area of language, Switzerland is divided in three geographic areas which correspond to three areas of languages: German, French, Italian, income, level of education and employment status. Moreover global health was measured through two items: perceived health (4 levels) and presence of longstanding illness. Information about the number of the visits with a family physician in the last 6 months was also collected (patient self-reported). Lastly, we also used as covariate the answer to the question: “Would you recommend this FP to a friend or a relative?” as a proxy of the patient‘s global satisfaction about the physician.

Family physicians’ data were collected via self-administrated questionnaire sent by postal mail. Socio-demographic features of FP were described in terms of sex, age, and rural/urban practices area. Questions also included general features (solo/group practice, FP in practice as unique activity), consultation length, weekly workload and care collaboration including workforce in the practice [22]. Exposure to work-related stress was explored through a proxy of the Siegrist’s effort/reward imbalance model (ERI) assessed using a single item, the following affirmation: “In my work there is a good balance between effort and reward” (strongly agree / agree/ disagree / strongly disagree then regrouped in “Exposure to ERI yes=agree + strongly agree /no=disagree + strongly disagree” for the statistical analysis) [23]. The ERI model claims that exposure to high efforts at work, both quantitative and qualitative demands, and inadequate rewards in return, in terms of money, esteem from colleagues or society, job security, can generate stress at work, with a potential impact both on physician’s well-being and performance [15, 16, 24].

Both physicians and patients’ questionnaires were elaborated in the three national languages of Switzerland: German, French and Italian language.

### Statistical analysis

The initial patient sample consisted of 1791 patients; for the present analysis, we excluded those who answered “I don’t know” to the question about enablement. However, we also performed a sensitivity analysis regrouping the “don’t know” with the “no” answers (versus “yes”). Continuous covariates such as patient and FP age, consultation length, and weekly workload, were dichotomized at their median.

The feeling of not being able to cope better with health problems, considered as the dependent variable, was analyzed using multilevel logistic regression (mixed logistic regression) models in order to take into account of the nested nature of the observations (9 patients in each practice). The variance explained at the practice level is reported at the end of the multivariate analysis table as “Practice variance”. As a first step, associations with sociodemographic, personal patients’ characteristics and FP characteristics were considered in simple logistic regression models. The variables associated with the outcome at a *p*-value of 0.2 or less were then included in a multiple model. Backward stepwise selection was performed to obtain a final model for patients’ characteristics. As a second step, association between enablement and each FP characteristic were separately studied using the final model selected for patients’ characteristics. The variables associated with the outcome at a *p*-value ≤0.2 were then included in the joint multiple logistic regression model. A manual backward stepwise selection (removal of the least significant variable at each step) with a *p*-value for selection set at 5% was performed to obtain a final model including both patients and FP characteristics. Analyses were performed using STATA software.

## Results

Table 1 describes the characteristics of the two samples. In the studied sample, 57% were female; the median age was 59 years, around 74% of the patients were born in Switzerland. Ninety percent of the patients declared that they coped better with their health problem just after the consultation. The patients were interviewed in 199 different practices. Physicians were mostly men (78%) with a median age of 56 years old. They worked in group practice in 52% and in urban area in 48%.

**Table 1:** Characteristics of the patients (*n* = 1463) and their family physicians (*n* = 199) in the sample

	N	Frequency (%)
Patients	1463	
Gender		
male	629	43.0
female	834	57.0
Age		
< 45	395	27.1
45–59	341	22.3
60–70	341	23.9
> 70	381	26.7
Language area		
German	857	58.5
French	423	28.9
Italian	183	12.5
Job seeking or invalidity		
Yes	136	9.3
No	1324	90.7
Income		
Below average	207	14.1
Around average	1091	74.6
Above average	165	11.3
Education level		
No qualification	459	31.3
Upper secondary	753	51.5
Post secondary	251	17.2
Country of birth		
Switzerland	1086	74.2
EU	255	17.5
Other	122	8.3
Main reason of the visit		
Ill or didn’t feel well	557	38.1
Other	906	61.9
Longstanding disease		
No	808	55.2
Yes	655	44.8
Perceived health		
Good or very good	984	67.3
Fair or poor	479	32.7
Patient’s own doctor		
No	93	6.4
Yes	1359	93.6
Would recommend this physician		
No	31	2.1
Yes	1432	97.9
Patient enablement		
Yes	1340	91.6
No	123	8.4
Family physicians	199	
Gender		
Male	155	77.9
Female	44	22.1
Age		
< 56	94	47.2
> =56	105	52.8
Practice area		
Rural	102	51.8
Urban	95	48.2
Group practice		
Yes	104	52.3
No	95	47.7

In the final multiple model including patient variables, a lower enablement was associated with younger age (OR = 1.64 [1.06–2.51]), French speaking area (OR = 1.67 [1.10–2.55]) (and German speaking when compared to Italian) and an above average income (1.80 [1.04–3.12]). An existing long standing disease and a poor perceived health were also associated with a lower enablement (resp. 1.58 [1.02–2.46] & 1.67 [1.07–2.62]). Lastly, the difficulty to cope better with health problems was reduced when the patients are satisfied with their doctor (recommend him) (OR = 0.21 [0.09–0.50]) and when the number of visits in the last 6 months was comprised between 1 and 4 (OR = 0.37 [0.22–0.61]) (Table 2, model 1).

**Table 2:** Patient characteristics associated with enablement (enablement = NO), in multilevel logistic regression

			Univariate analyses		Multivariate analyses (model 1, *n* = 1428)	
	n	%	OR	CI 95%	OR	CI 95%
Gender (ref Female)						
Male	629	43.0	1.39	[0.95–2.03]	1.26	[0.85–1.88]
Age (ref > = 60)						
< 60	736	50.3	1.66	[1.13–2.45]	1.64	[1.06–2.51]
Language area (ref: German)						
French	423	28.9	1.89	[1.29–2.77]	1.67	[1.10–2.55]
Italian	183	12.5	0.21	[0.06–0.66]	0.22	[0.07–0.74]
Job status (ref. Employed, retired, student)						
Job seeking or invalidity	136	9.3	2.01	[1.18–3.41]	–	–
Country of birth (ref: Switzerland)						
Other	378	25.8	0.85	[0.54–1.33]	–	–
Education level (ref: No qualification)						
Upper secondary	754	45.8	1.02	[0.67–1.56]	–	–
Post secondary	251	17.1	0.80	[0.44–1.45]	–	–
Income around average (ref: Around average)						
Below average	207	14.1	1.50	[0.90–2.49]	1.11	[0.64–1.92]
Above average	165	11.3	1.85	[1.10–3.13]	1.80	[1.04–3.12]
Main reason of the visit (ref: Other)						–
Ill or did not feeling well	558	38.1	1.00	[0.67–1.47]		
Longstanding disease (ref. No)						
Yes	656	44.8	1.47	[1.00–2.16]	1.58	[1.02–2.46]
Perceived health (ref. Very good or good)						
Fair or poor	480	32.8	1.73	[1.18–2.55]	1.67	[1.07–2.62]
Patient’s own doctor (ref. No)						
Yes	1359	93.7	0.48	[0.26–0.91]	0.57	[0.30–1.11]
Recommend this doctor (ref. No)						
Yes	1432	97.9	0.20	[0.08–0.46]	0.21	[0.09–0.50]
Number of visits in the last 6 months (ref: 0)						
1–4 times	839	57.3	0.40	[0.25–0.65]	0.37	[0.22–0.61]
> 4 times	354	24.2	0.80	[0.48–1.34]	0.60	[0.34–1.06]
Practice variance*					0.07	

*Explained variance at the practice level

The associations between the physicians’ characteristics and patient enablement were then studied separately one by one adjusting on the selected patients variables (Table 3, model 2). Patient difficulty to cope better with health problems was lower among the physicians exposed to the effort reward imbalance (OR (for enablement = no): 0.63 [0.40–0.99]). FP gender (*p* =0.2) and the presence of other paramedical disciplines in the practice (*p*= 0.1) were also retained in the final model both including patients and physicians’ characteristics (Table 3, model 3). In the final model, the association between patient enablement and physician’s exposure to an effort-reward imbalance was borderline significant (OR (for enablement = no): 0.64 [0.41–1.00]). The sensitivity analysis, regrouping people who answered “I don’t know” with people having answered “no” to the question of coping better after a medical visit, reported close results (not shown). Finally, the variance explained at the practice level was small (0.04), meaning that most of the determinants of patient enablement are patient-based.

**Table 3:** Patients and physicians (or practice) characteristics associations with patients enablement (enablement = NO) in multilevel logistic regressions

			Model 2		Model 3 (*n* = 1412)	
	n	%	OR	CI 95%	OR	CI95%
Patientsc’ characteristics						
Age (ref > = 60)						
< 60					1.58	[1.03–2.41]
Language area (ref: German)						
French					1.60	[1.04–2.44]
Italian					0.20	[0.06–0.65]
Longstanding disease (ref. No)						
Yes					1.54	[1.00–2.39]
Perceived health (ref. Very good or good)						
Fair or poor					1.62	[1.04–2.51]
Patient’s own doctor (ref. No)						
Yes					0.51	[0.26–0.98]
Recommend this doctor (ref. No)						
Yes					0.18	[0.08–0.44]
Number of visits in the last 6 months (ref: 0)						
1–4 times					0.37	[0.23–0.62]
> 4 times					0.64	[0.36–1.13]
Physicians’ characteristics (*n* = 199)						
GP Gender (ref. Male)						
Female	44	22.1	1.22	[0.76–1.96]		
GP Age (ref. < 56)						
> = 56			0.90	[0.60–1.34]	–	–
Practice area (ref: Urban)						
Rural area	95	48.2	1.13	[0.75–1.71]	–	–
Group practice (ref. No)						
Yes	104	52.3	1.07	[0.71–1.61]	–	–
GP as unique activity **(**ref. No)					–	–
Yes	67	33.7	1.07	[0.70–1.64]		
Consultation’s length (ref. < 20 min)				-	–	–
> = 20 min			1.03	[0.66–1.60]		
Weekly workload (ref. < 47 h)					–	–
> = 47 h			0.81	[0.53–1.22]		
Effort reward imbalance exposure (ref. No)						
Yes	70	31.3	0.63	[0.40–0.99]	0.64	[0.41–1.00]
Other paramedical disciplines in the practice (ref. No)						
Yes	25	12.6	1.51	[0.90–2.52]		
Practice Variance*					0.04	

Models 2: Physicians’ characteristics separately adjusted with patients characteristics in final model 1- Model 3: Multivariate final model including patients and physicians characteristics

*Explained variance at the practice level

## Discussion

This study first demonstrates that only a small minority of patients feel they cannot cope better with their health problems directly after a FP visit. The study also highlights that the patients’ personal characteristics and factors related to the patient-physician relationship are the most important predictors of that feeling. Enablement is indeed associated with patient age, linguistic area in Switzerland, chronic disease, the number of FP’s visits during the last 6 months, and the satisfaction with his physician. Finally, job stress among physicians, assessed through the effort-reward imbalance, is borderline associated with lower difficulties to cope better with health problems among patients.

### Comparison with the literature

Heterogeneity in patient enablement was observed between linguistic areas through the country, while we found no association with the country of birth. Some studies already reported links with ethnicity or language skills but it is not the same background as leaving in a country with three main languages and three main cultures [7, 9]. This result highlights the potential importance of the cultural aspect attached to that concept. Such discrepancies could have different origins: interpretation of the question according to the linguistic area, different expectations regarding family physicians, and finally, a different patients-physician relationship. More qualitative research would be needed to understand the mechanism underlying this result.

Our results regarding the association with patients’ age are consistent with those reporting by Mead who found that patient in middle age groups (31–60 years) reported lower enablement [7]. In our study, we divided the patients into two groups according to the median age of 59 years old and those in the “young” group were mainly 30–60 years old. Even if expectations and experience may vary throughout life, we can also make the hypothesis that a longer relation between the patient and physician (which is generally the case for older patients) play a role in better patient enablement. Moreover, the patient difficulty to cope with health problems is about lower among patients visiting their own doctor. The association between enablement and the frequency of visits during the last 6 months suggests that there might be an “ideal” number of visits for a better enablement, not too few but not too many. This supports the importance of the patient-physician relationship, not only considering its duration but also the frequency of encounters. However, we can also state that we found a lower ability to cope with health troubles among patients with low frequency of encounters because they do not reconsult the same FP. These results can be underpinned by the already reported positive association between continuity of care and patient enablement [11, 14]. Furthermore a high number of visits, in cases of patients with chronic diseases (probably leading to higher continuity of care) is not associated with less difficulties for the patient to cope better with health problems. Indeed the association between poor enablement and longstanding illness or poor perceived health has been founded in several studies [7, 14] as also observed in our data. And finally note also that the relationship between the patient enablement and number of visits persists after adjusting for existing chronic disease and perceived health. The question of the frequency of patient visits is an important issue in family medicine; does a higher frequency of visits guarantee better health outcomes? Setting the right timing for patients’ visits is indeed one of the biggest challenges in family medicine, not answered by textbooks and difficult to teach to residents. Our results suggest that it is not the case for patient enablement. This issue may also have to do with the concept of the therapeutic alliance which is a component of the patient-centred medicine. Such alliance, based upon the relationship between the physician and his patient “has potential therapeutic benefit in and of itself” [25]. There was a strong association between recommending his doctor and patient difficulty to cope with his health problems (OR = 0.18). We chose to include this factor as a global measure of the patients’ satisfaction with their family physician. The relationship between enablement and satisfaction in the literature is not clear; they are close and inter-related but distinguished concepts [21]. Thus, the intensity of this association is not surprising but could also reflect the circularity of the data and may be due to a common-method bias (dependant and independent variables both collected simultaneously using same method) [26]. That is also why we chose not to include other subjective items related to the patient experience (with the exception of a global proxy of satisfaction) about their visit and we chose to focus on others characteristics related to physicians. Lastly, the inclusion of this variable did not change the results about the other variables of the final model.

The unique association (actually borderline significant) with physician characteristics is related to a job stress factor i.e. the exposure to the effort-reward imbalance. This finding is surprising and quite difficult to interpret. Indeed we expected that physician’s exposure to ERI would have increased the difficulties of coping better with health problems among the patients. We could hypothesize, first, that the high efforts provided by the physicians could be perceived by the patients as a good implication, and secondly, that the potential resulting stress for the physicians could possibly not be perceived by the patients. This is a very cautious assumption which should be considered as such. Qualitative researches could be helpful for a better understanding.

We found no association between enablement and the length of the consultation. This association has been described in some studies [10]. Two reasons could explain such discrepancies. First in our study, the consultation length was a physician‘s variable, defined as the mean duration of his consultation. It was not an individual variable declared by the patient in link with the visit he just had. Secondly, average time of consultation in Switzerland is already one of the longest (around 19 min [22]) as compared to other industrialized countries like Great Britain for instance. Here the mode of remuneration of the physicians (Switzerland is a fee for time and service system) may probably be taken into account to study this relationship.

Regarding organizational practice characteristics, one of our hypotheses was that working alone or with other physicians or paramedical disciplines could play a role in patient enablement, either positively through improved coordination, continuity, or in a deleterious way by fragmenting care. We found no association between enablement and group practice. Regarding the other paramedical disciplines in the practice the results are less clear with an association which does not remain in the multiple final model. This result suggests, at least, that the difficulties to cope better with their health troubles for the patients are not reduced by the presence of a large medical team in the practices. However, models of interdisciplinary care are not common in Switzerland and future research would probably be useful to explore this issue, particularly in countries with other models of care organization.

### Limitations and strengths

This study has several limitations. The representativeness of both samples might be limited. Despite random sampling and a good representativeness in terms of age, gender and rural/urban repartition among physicians, the low participation rate (about 10%), although classically observed in such practice-based research network, might introduce some level of bias on other unmeasured characteristics. Among patients, the participation rate was high (around 84%) but we cannot exclude a selection of some patients more able to cope better with their health trouble. Nevertheless, the lack of representativeness is less problematic in analytic analyses, than in descriptive ones. A small sample size, particularly regarding physicians’ data, probably limits the possibility to observe more significant associations. Indeed, using multilevel regression models, which is appropriate considering the nature of the data, generates a less favorable situation to obtain statistically significant results. We can also mention the use of a proxy to assess enablement instead of a standardized instrument such as the Patient Enablement Instrument, PEI [4], but the chosen question constitutes the core of the concept of enablement.. Lastly, this is a cross-sectional study which does not allow affirming the causal nature of the associations.

The main strength of this study is the richness and the quality of the data. They were collected from international standardized protocols and questionnaires and we have data both from patients and physicians. This design allows a multilevel analysis which provides an additional asset.

## Conclusion

This study showed that the predictors of patient enablement seem to be mainly centered on his/her own characteristics and the relationship with his/her physician. Organizational practice characteristics do not seem to play a major role in patient enablement, but Swiss practices are rather small and relatively homogenous in their functioning. Despite the design of the study that does not allow causal inference our results confirm previous findings suggesting that, FPs should pay particular attention in their relationship with young patients and those with longstanding disease or poor perceived health for a better patient enablement. The continuity of the patient-physician relationship, both including frequency of the visits and satisfaction of the patient could also be considered in order to optimize the potential direct benefit from their relationship. The findings regarding the potential influence of exposure to job stress must be further investigated. In such context including numerous subjective factors, quantitative analyses are limited to explore the mechanisms and should be complemented by qualitative analyses.

## Abbreviations

ERI: Effort/reward imbalance model
FP: Family physicians
OR: Odds ratio
PEI: Patient enablement instrument
PROM: Patient-reported outcome measure
QUALICOPC: Quality and costs of primary care
